# The Impact of Perspective Change As a Cognitive Reappraisal Strategy on Affect: A Systematic Review

**DOI:** 10.3389/fpsyg.2016.01715

**Published:** 2016-11-04

**Authors:** Sophie M. A. Wallace-Hadrill, Sunjeev K. Kamboj

**Affiliations:** ^1^East London National Health Service Foundation TrustLondon, UK; ^2^Research Department of Clinical, Educational and Health Psychology, University College LondonLondon, UK

**Keywords:** vantage perspective, mental imagery, affect, reappraisal, semantic change

## Abstract

The strategic or *deliberate* adoption of a cognitively distanced, third-person perspective is proposed to adaptively regulate emotions. However, studies of psychological disorders suggest *spontaneous* adoption of a third-person perspective reflects counter-productive avoidance. Here, we review studies that investigate the deliberate adoption of a third- or first-person vantage perspective and its impact on affect in healthy people, “sub-clinical” populations and those with psychological disorders. A systematic search was conducted across four databases. After exclusion criteria were applied, 38 studies were identified that investigated the impact of both imagery and verbal instructions designed to encourage adoption of a third-person perspective on self-reported affect. The identified studies examined a variety of outcomes related to recalling memories, imagining scenarios and mood induction. These were associated with specific negative emotions or mood states (dysphoria/sadness, anxiety, anger), mixed or neutral affect autobiographical memories, and self-conscious affect (e.g., guilt). Engaging a third-person perspective was generally associated with a reduction in the intensity of positive and negative affect. Studies that included measures of semantic change, suggested that this is a key mediator in reduction of affect following perspective change. Strategically adopting a “distanced,” third-person perspective is linked to a reduction in affect intensity across valence, but in addition has the potential to introduce new information that regulates emotion via semantic change. Such reappraisal distinguishes deliberate adoption of a distanced perspective from the habitual and/or spontaneous shift in perspective that occurs in psychopathology.

## Introduction

The use of cognitive strategies to modulate emotions is critical for adaptive self-regulation. Assuming a generative role for appraisals in the experience of emotion, the dampening and intensifying of emotion can be achieved by altering the *meaning* of preceding situations or events. Emotion-regulation via “reappraisal” is achieved through two neurally separable and psychologically distinct routes (Ochsner and Gross, 2008). Firstly, information can be verbally reinterpreted, such that a situation or stimulus is regarded as less threatening. This is how the term reappraisal has traditionally been used, for example in cognitive behavioral therapy. Secondly, a third-person, detached or “distanced” perspective can be employed as a form of reappraisal; this can produce similar affect-regulating effects to verbal strategies (Ochsner and Gross, 2008)^1^. Such perspective-shifting might be conducive to changes in *meaning* by enabling the individual to disengage from a self-immersed vantage point and “see the bigger picture.”

Importantly, as well as visuospatial (imagery) strategies, a distanced perspective can be achieved through verbal means, by switching from first-person (“I”) to third-person (“she/he”) pronoun-use (or by using proper nouns; Kross et al., 2014) when describing the self. In either case, perspective change-strategies permit the introduction of new and regulatory information during appraisal of situations or events. This is important because a variety of psychological disorders are associated with a narrowing of information-processing “bandwidth” (cognitive biases; Mathews and MacLeod, 2005) and a tendency to spontaneously and habitually experience memories or simulated future events from first- or third-person perspectives. In these instances, deliberately-employed perspective-shifting strategies may have an adaptive, emotion-regulating effect, with implications for their use in therapeutic and normative contexts.

The phenomenology of the visual vantage perspective has been examined in various psychological disorders during instructed recall of autobiographical events. For example, adults and adolescents with social phobia tend to experience imagery of social situations from a third-person perspective (Hackmann et al., 1998, 2000; Wells et al., 1998; D'Argembeau et al., 2006; Schreiber and Steil, 2013). Indeed, a distorted third-person self-image is argued to be a key maintaining factor within the cognitive model of social phobia (Clark and Wells, 1995). Higher degrees of anxiety appear to be linked to increased third-person perspective in social phobia (Coles et al., 2001), which also comes to dominate social phobia-related memories over time (Coles et al., 2002).

Other anxiety disorders, including agoraphobia (Wells and Papageorgiou, 1999; Day et al., 2004) and body-dysmorphic disorder (Osman et al., 2004) share a similar tendency for memories to be recalled from a third-person perspective. By contrast, people with obsessive compulsive disorder (OCD) report more first-person autobiographical memories (Lipton et al., 2010), and more first-person images of dirt and contamination situations compared to a non-clinical control group (Coughtrey et al., 2013).

Posttraumatic Stress Disorder (PTSD) symptoms are linked to increased third-person perspective recall of trauma memories (Berntsen et al., 2003). In contrast to OCD and social phobia, trauma-related memories *deliberately* recalled from this perspective are reported to be *less* affect-provoking (McIsaac and Eich, 2004). On the other hand, higher levels of avoidance have been linked to an increased incidence of *spontaneous* (i.e., intrusive) third-person perspective trauma memories (Kenny and Bryant, 2007), and occurrence of third-person perspective during deliberate recall predicts severity of PTSD symptoms up to 1 year after the traumatic event (Kenny et al., 2009). These findings suggest that the tendency to adopt a third-person perspective during explicitly cued or spontaneous recall is an avoidance strategy that may play a role in maintenance of PTSD.

Studies that have reported an association between depression symptoms and perspective suggest that memory valence and perspective interact. For example, recall of negative memories from a third-person-perspective in depressed patients is linked to higher use of maladaptive avoidant strategies such as “emotional detachment” and rumination (Lemogne et al., 2006; Williams and Moulds, 2007; Kuyken and Moulds, 2009). In addition however, vulnerability to depression appears to be linked to deficits in first-person *positive* memories (Lemogne et al., 2006; Bergouignan et al., 2008; Nelis et al., 2013). Relatedly, although negative memories recalled from a first-person perspective were linked to increased distress, only *positive* memories recalled from a third-person perspective were linked to experiential avoidance (Moulds et al., 2012).

Overall, the association between third-person perspective and psychological symptoms or negative affective states across a range of psychological disorders outlined above might suggest that adoption of this perspective is a maladaptive (avoidance) emotion-regulation strategy rather than an adaptive reappraisal strategy (see e.g., Williams and Moulds, 2007). However, since the studies on psychopathology and perspective outlined above are largely correlational, causal inferences are not possible. As such, experimental studies examining the link between affect change through memory recall, imagining scenarios and mood induction (a potential model for relevant symptoms of psychopathology) and perspective (change) through verbal or imagery-manipulation are particularly valuable in determining the role of perspective in psychological disorder and emotion regulation. Moreover experimental manipulation of perspective allows the sufficiency of a third-person perspective in emotion regulation to be tested. Specifically, it may be that adoption of such a distanced perspective sets the stage for reappraisal but in isolation has limited effects on emotion. As such, experimental studies will be the focus of the remainder of this review.

## Methods

A systematic review of the literature was conducted using four databases, PsychInfo, Embase, Medline, and Web of Science. The time-frame was limited from 1980 to 2014. The search was conducted on 7th Oct 2014 using terms relating to perspective, affective descriptors and either mental imagery or verbal strategies (Appendix 1).

Inclusion criteria for the review were (i) articles in peer-reviewed journal, (ii) published in English, (iii) relating to adults, (iv) experimental studies in which (v) participants were assigned to one of two vantage perspective conditions (first- or third-person) using a randomized or quasi-experimental design and reported (vi) at least one affect-related outcome measure. Note, our use of the term “vantage perspective” does not imply adopting and understanding *another's* perspective (i.e., theory of mind).

**Figure 1:**
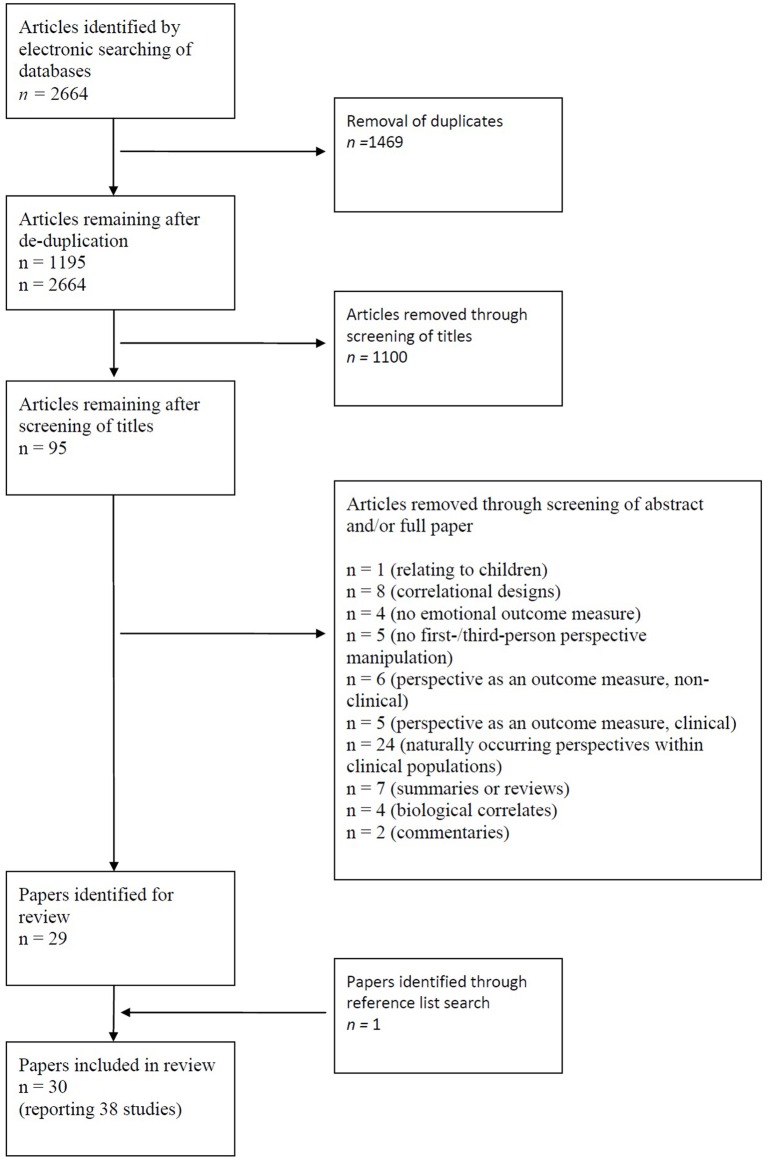
**Flowchart of literature search process**.

The initial search produced 2664 articles, and 1469 duplicates were removed, leaving 1195 articles. Titles, and where necessary abstracts for clarification, were reviewed to establish subject and category relevance (excluding e.g., articles from chemistry journals and other life sciences), leaving 95 articles. An abstract and full article text search was conducted for all 95 articles, and 37 studies from 29 articles were identified for review. References lists were also searched; one additional article was identified yielding 38 studies in total (see Figure 1).

## Results

The 38 studies identified in the search investigated the impact of perspective on affect following memory recall, induction of mood or imagining various scenarios (Supplementary Table 1). For the purposes of this review, studies are organized into six broad categories based on affect/mood type (including clinical disorders typically associated with these affects, where relevant). Participants comprised healthy controls, sub-clinical populations and clinical populations. As such, the included studies were of (i) *sadness* in non-clinical or “sub-clinical” (dysphoric) participants, and those with diagnostically-verified depression, (ii) *anxiety* in non-clinical participants and people with sub-clinical symptoms (iii) *anger* in non-clinical participants, (iv) *self-conscious emotions* (e.g., guilt) in non-clinical participants, (v) *mixed or neutral affect* in non-clinical participants, and finally (vi) *positive emotions* in clinical and healthy populations.

### Sadness/dysphoria

All five identified studies examined the effect of perspective on autobiographical memories associated with sadness (Kross and Ayduk, 2008; Williams and Moulds, 2008; Grisham et al., 2011; Wisco and Nolen-Hoeksema, 2011; Kross et al., 2012).

#### Sample and methodological characteristics in sadness/dysphoria studies

Non-clinical samples were used in four studies, and variously examined memories of “overwhelming sadness and depression” (Kross and Ayduk, 2008) and “sad” experiences (Grisham et al., 2011). Kross and colleagues investigated memories of a “depressing life experience” in non-clinical participants (Kross et al., 2012), but also examine depressed patients, the only relevant study identified that examined a clinical group (see Supplementary Table 1).

Williams and Moulds (2008) also used non-clinical participants. Although they were selected only on the basis of the occurrence of involuntary memories and not the presence of symptoms of depression, their sample was described as “mildly dysphoric.” However, scores on the Beck Depression Inventory-II (BDI-II; Beck et al., 1996) were typical of healthy volunteers, suggesting that the term dysphoric may not have been appropriate for their sample (Wang and Gorenstein, 2013). In contrast, Wisco and Nolen-Hoeksema (2011) purposively recruited sub-clinical participants from a student and community sample based on high or low scores on the BDI-II (≥16 and ≤ 9, respectively).

#### Effects of perspective on emotion in sadness/dysphoria studies

Studies generally demonstrate a link between the deliberate adoption of the third- vs. first-person perspective and a reduction in negative affect and related outcomes (Kross and Ayduk, 2008; Grisham et al., 2011; Wisco and Nolen-Hoeksema, 2011; Kross et al., 2012; see Supplementary Table 1 for more details). Two studies suggested a dependence of the perspective-affect relationship on depressive symptomatology (Williams and Moulds, 2008; Kross et al., 2012), although others found that the relationship holds even in the absence of depression or dysphoria (Kross and Ayduk, 2008; Grisham et al., 2011; Wisco and Nolen-Hoeksema, 2011). Anxiety was also reduced when a negative intrusive memory was re-recalled from the third-person perspective, and vice versa for the alternative perspective (Williams and Moulds, 2008).

#### Additional reappraisal-related measures in sadness/dysphoria studies

Kross and colleagues assessed semantic differences in participants' memories of sadness and depression (Kross and Ayduk, 2008). Greater reconstrual (a change in meaning or understanding about a situation) was found in participants instructed to recall from the third-person perspective, and mediated the effect of perspective on affect (Kross and Ayduk, 2008).

### Anxiety/threat

Supplementary Table 1 outlines six studies that examined the effects of perspective shifting during induced anxiety or threat (Spurr and Stopa, 2003; Lau et al., 2009; Wang et al., 2012; Kross et al., 2014).

#### Sample and methodological characteristics in anxiety/threat studies

All studies induced anxiety/threat experimentally; five studies tested non-clinical (Lau et al., 2009; Wang et al., 2012; Kross et al., 2014) and one (Spurr and Stopa, 2003) sub-clinical (and control) samples based on high and low scores on the Fear of Negative Evaluation Scale (FNES; Watson and Friend, 1969).

#### Effects of perspective on emotion in anxiety/threat studies

Reductions in anxiety and negative emotions were seen in most studies instructing participants to adopt a third- vs. first-person perspective (Wang et al., 2012; Kross et al., 2014). However, a study comparing participants with sub-clinical social anxiety with non-anxious controls, did not find a statistically significant reduction in anxiety following third-person perspective instructions while recalling a anxiety-provoking performance (Spurr and Stopa, 2003).

The experience of negative social evaluation appeared to be linked to the third-person perspective in an ostracism paradigm among non-clinical participants. For deliberately “excluded” participants, those instructed to adopt a third-person perspective when recalling their experience reported an increased perception of threat over time (Lau et al., 2009). No differences were found for the “included” participants, regardless of perspective.

When attachment styles were considered (Wang et al., 2012), a third-person perspective reduced negative affect in those with low, but not high scores on a measure of avoidant attachment. However, both low and high anxious attachment scorers showed lower levels of negative affect when writing in a third-person perspective.

#### Additional reappraisal-related measures in anxiety/threat studies

As in previous studies, Kross et al. (2014) included a post-event semantic-processing measure (“stream of thought” essay), either coded for recounting and reconstruing (Study 3) or challenge and threat appraisals (Study 4). The third-person perspective was associated with more reconstruing and greater perception of the task as a challenge rather than a threat, indicating that the use of this perspective encouraged an adaptive shift in understanding or appraisal of the situation.

#### Methodological limitations in anxiety/threat studies

Although Spurr and Stopa (2003) used a number of validated measures of anxiety in their study, the cognitive load associated with performing the first- and third-person perspective conditions was not well-matched. In particular, the third-person condition required performing a secondary speech-task whereas the first-person condition involved a relatively simple external focus (“try as much as possible to be aware of the environment rather than of yourself,” p.1017), which likely entailed less cognitive load.

### Anger

Six studies relating to anger experiences were identified (Kross et al., 2005; Ayduk and Kross, 2008; Ray et al., 2008; Wimalaweera and Moulds, 2008; Mischkowski et al., 2012). These studies are characterized by the use of “why” (rather than “what” manipulations) which encouraged participants to consider the causes and context of the event (rather than merely the events themselves).

#### Sample and methodological characteristics in anger studies

All studies were with non-clinical participants. Five studies asked participants to recall an experience of feeling anger (Kross et al., 2005; Ayduk and Kross, 2008; Ray et al., 2008; Wimalaweera and Moulds, 2008). One study (Mischkowski et al., 2012) induced anger experimentally using a provocation task (Bushman et al., 2005).

#### Effects on emotion in anger studies

The third-person perspective was associated with lower levels of anger, emotional reactivity and negative affect in five studies, particularly when a “why” focus was adopted (Kross et al., 2005; Ayduk and Kross, 2008; Ray et al., 2008; Mischkowski et al., 2012). One study (Wimalaweera and Moulds, 2008), however, failed to replicate the latter pattern, finding instead that the third-person “why” condition *increased* anger, along with increased negative affect and intrusions.

#### Additional reappraisal-related measures in anger studies

In their second study, Kross et al. (2005) also measured the effects of perspective on concrete and abstract construals of anger memories. They found that lower levels of concrete (relative to abstract) understanding mediated the third-person “why” effect, i.e., greater abstract processing was a key element in affect reduction when participants considered situations using a “why analysis.”

#### Methodological limitations in anger studies

Kross et al. (2005) assessed affect following recall of anger memories, but did not examine whether state anger/negative affect or the valence of the memories themselves were equivalent at baseline, a limitation that was addressed in later study by Wimalaweera and Moulds (2008). However, this latter study, which did not replicate Kross et al.'s (2005) findings, may have been insufficiently powered (~*n* = 15 per condition) to detect a medium effect (Ayduk and Kross, 2009).

### Self-conscious emotions

Three publications reporting seven studies relating to self-conscious emotions were identified (Libby and Eibach, 2011; Hung and Mukhopadhyay, 2012; Katzir and Eyal, 2013). Self-conscious emotions, e.g., guilt and shame, can be defined as those involving self-reflection, self-evaluation, and involving some form of “falling-short” in relation to personally important standards of behavior (Tracy and Robins, 2007). Self-conscious emotions are linked to inferences about how others may perceive and evaluate the self (Leary, 2007). For example, guilt is a response to specific behavioral transgressions, whereas shame has been linked to actions which reflect negatively on a person's entire character (Tangney and Dearing, 2003). This is in contrast to more global negative self-evaluations often associated with depressed mood and anxiety.

#### Sample and methodological characteristics in self-conscious emotion studies

All studies were with non-clinical participants. Two studies asked participants to imagine novel scenarios in which they might be expected to feel self-conscious emotions (e.g., embarrassment following a socially exposing imagined situation) (Hung and Mukhopadhyay, 2012, Studies 1 and 3) and one study examined recall of memories of resisting or succumbing to temptation, both potentially socially-evaluative situations (Hung and Mukhopadhyay, 2012, Study 2). Two studies (both in Katzir and Eyal, 2013) compared memories of self-conscious emotions (shame/guilt) to basic emotions (sadness/anger) and two examined the relationship between either imagined scenarios or memories of failure experiences, shame, and perspective in participants with high and low self-esteem (Libby et al., 2011).

#### Affect outcomes in self-conscious emotion studies

A number of studies found that instructions to imagine or recall scenarios and events using a third-person perspective were associated with higher self-conscious affect. A third-person perspective also increases *positive* self-conscious emotions (e.g., pride) when “resisting temptation,” and negative self-conscious emotions (e.g., guilt) when “succumbing to temptation” (Hung and Mukhopadhyay, 2012, Studies 1 and 2). A similar pattern was seen in an imaginary scenario linked to both excitement and embarrassment (Hung and Mukhopadhyay, 2012, Study 3) in which a third-person perspective increased embarrassment, while simultaneously lowering levels of excitement. Self-esteem mediated the effect of third-person perspective on shame in failure memories such only that those with lower self-esteem experienced higher shame from a third-person perspective (Libby et al., 2011).

However, in two studies (Katzir and Eyal, 2013), the third-person perspective condition was not associated with increased levels of self-conscious emotion (guilt and shame), but was associated with a decrease in anger and sadness, replicating previous studies (Kross et al., 2005; Kross and Ayduk, 2008).

#### Additional reappraisal-related measures in self-conscious emotion studies

In their second and third studies, Hung and Mukhopadhyay (2012) also included measures of appraisals of the autobiographical memories; those using a third-person perspective thought more about how others might evaluate them rather than the positive aspects of the experience. This was found to mediate the effect of perspective on affect in both studies.

Katzir and Eyal (2013) also included a written task of the anger/guilt, and sadness/shame memory which was independently coded for self-evaluations. Although they found that self-evaluations were more prevalent in the self-conscious emotion condition compared to the “basic” emotion condition, there was no effect of perspective, suggesting that these appraisals did not mediate the effect of the third-person perspective.

#### Methodological limitations in self-conscious emotion studies

The outcome measures assessed by Hung and Mukhopadhyay (2012) do not differentiate between “guilt” and “shame” (Tangney and Dearing, 2003). In all three studies only a measure of guilt is used. It is possible that inclusion of a measure of shame would have further clarified the impact of perspective change.

### Studies on mixed and neutral affect autobiographical and episodic memory in non-clinical samples

Nine studies were identified relating to autobiographical/episodic memory (Robinson and Swanson, 1993; Berntsen and Rubin, 2006; Terry and Horton, 2007; Bagri and Jones, 2009; Crawley, 2010; Sutin and Robins, 2010; Seih et al., 2011; Sekiguchi and Nonaka, 2014).

#### Sample and methodological characteristics in mixed and neutral affect autobiographical and episodic memory studies

All studies were with non-clinical samples. Three studies investigated “negative” autobiographical memories (Terry and Horton, 2007; Crawley, 2010; Seih et al., 2011). Two studies investigated both negative and “positive” autobiographical memories (Berntsen and Rubin, 2006; Sekiguchi and Nonaka, 2014). Two studies investigated autobiographical memories without specifying valence to participants (Robinson and Swanson, 1993; Sutin and Robins, 2010), although one asked participants to recall “self-defining” autobiographical memories (Sutin and Robins, 2010).

Two studies were identified which investigated recall of experimentally presented material of fictional scenes (Bagri and Jones, 2009).

#### Affect outcomes in mixed and neutral affect autobiographical and episodic memory studies

One study found reduced levels of overall emotion and “nervousness” from a third-person perspective (Terry and Horton, 2007) and third-person recall was associated with reduced emotional involvement and emotional intensity in two writing studies (Crawley, 2010; Seih et al., 2011). Recall of affective material in episodic memory tasks was lower in a third-person condition; in an initial study there was no difference in the reported “emotional richness” of recall, but in a second, potentially more highly powered study, this was lower in the third-person perspective (Bagri and Jones, 2009). However, two studies found no link between the adoption of a third-person perspective and emotional intensity (Berntsen and Rubin, 2006; Sutin and Robins, 2010).

The original (i.e., spontaneous) perspective of a memory appears to have an important role in the effect of using a third- or first-person perspective. In three studies, levels of affect decreased only when first-person memories were recalled from the third-person, and not vice versa (Robinson and Swanson, 1993; Berntsen and Rubin, 2006; Sekiguchi and Nonaka, 2014). This implies that shifting from a third- to first-person memory does not intensify affect during recall.

#### Additional reappraisal-related measures in mixed and neutral affect autobiographical and episodic memory studies

Seih et al. (2011) also assessed use of cognitive mechanism words (e.g., “understand”) as a measure of cognitive processing, and found that the third-person perspective had lower levels of cognitive processing. This appears to contrast studies such as those reviewed above (e.g., Kross et al., 2014) which have linked the third-person perspective to *increased* “semantic” processing, arguably a related construct. This discrepancy may be explained by the instructions given to participants in Seih et al.'s study, i.e., a focus on “what” occurred rather than specifying a focus on “why.” In other studies, only the “why” focused third-person perspective shows increased semantic processing (e.g., Kross et al., 2005).

#### Methodological limitations in mixed and neutral affect autobiographical and episodic memory studies

Sutin and Robins (2010) did not find a difference in affect when perspective was manipulated. However, inspection of the reported means within the paper suggests that the mean emotional intensity of the manipulated first-person perspective is statistically lower than the spontaneously adopted (at recall) first-person perspective [*t*_(461)_ = 3.26, *p* = 0.001]. This anomaly was not discussed by the authors.

The sample size used in Seih et al. (2011) was relatively small per condition (~*n* = 18), thus their study may not have had sufficient power to detect smaller effect-size reductions in negative affect.

### Positive affect

Four studies relating exclusively to positive memories or imagined positive scenarios were identified (Holmes et al., 2008; Gruber et al., 2009; Nelis et al., 2012; Vella and Moulds, 2014).

#### Sample and methodological characteristics in positive emotion studies

Two studies investigated experimentally-presented positive scenarios in non-clinical samples (Holmes et al., 2008; Nelis et al., 2012). Despite focusing on positive emotions, both studies administered clinically relevant measures, the BDI-II (Beck et al., 1996) and the State Trait Anxiety Inventory trait scale (STAI-T; Spielberger et al., 1983) to establish levels of depressive and anxious symptomatology within their sample. One study with a non-clinical sample (Vella and Moulds, 2014) investigated positive memories and imagined positive future events.

One study (Gruber et al., 2009) compared the effect of changing perspective on memories of intense happiness using both a healthy control group and a euthymic group with bipolar I disorder, a condition associated with elevated mood (American Psychiatric Association, 2013).

#### Affect outcomes in positive emotion studies

A decrease in positive affect was linked to a third-person perspective in two studies, in both clinical and non-clinical participants (Holmes et al., 2008; Gruber et al., 2009). This positive affect reduction in the third-person perspective group was not replicated in another study (Nelis et al., 2012), in which there was no difference between the two imagery perspectives, both of which increased positive affect compared to general (non-perspective related) verbal processing. Shifting from the first- to third-person perspective for both positive memories and future imagined positive events, decreased positive emotions such as happiness, whereas the converse shift had no impact (Vella and Moulds, 2014).

#### Methodological limitations in positive emotion studies

Subsequent research (Nelis et al., 2013) has shown dysphoria is associated with increased use of third-person perspective in positive memories. Thus, it is possible that the first-person condition in the authors' earlier study (Nelis et al., 2012), with higher levels of depressive symptomatology, may have had a greater tendency to initially experience scenarios from the third-person, even if they then followed first-person instructions. Research reviewed above in autobiographical memories suggests that there is no reduction in affect when moving from a third- to a first-person perspective. This may explain the lack of difference between the conditions.

## Discussion

The focus of this review was on experimental studies that examined the effect of deliberate adoption of certain vantage perspectives on affect. The majority of studies were with healthy volunteers and as such, the findings are principally relevant to normative emotion regulation, with potential implications for psychopathology. Overall, the identified studies tended to show that, compared to a first-person perspective, instructions to adopt a third-person perspective was associated with reduced negative *and* positive affect, a pattern also observed in the small number of studies with clinical participants.

Several studies investigated the affective impact of perspective during recall of sad or depressive experiences. The studies reviewed in this paper indicate that for both non-clinical participants, and those with subclinical and clinical depressive symptoms, strategic (i.e., “instructed”) adoption of the third-person perspective when recalling upsetting memories is generally linked to lower negative affect or emotional intensity (see Supplementary Table 1). This would appear to indicate that the deliberate use of the third-person perspective during recall of distressing memories activates top-down cognitive control processes resulting in effective emotion-regulation. In contrast *preferential* (“non-instructed” or spontaneous) adoption of the third-person perspective is linked to dysfunctional avoidance of distress during voluntary recall of negative and positive experiences in those with depression or a vulnerability to depression (Lemogne et al., 2006; Williams and Moulds, 2007; Kuyken and Moulds, 2009).

In line with the above, studies relating to anxious and threat-based memories in healthy volunteers tended to show that instructions to adopt a third-person perspective were linked to lower negative affect and anxiety. This contrasts with clinical or sub-clinical anxiety, which is associated with a spontaneous bias toward third-person perspective during recall of anxiety-provoking situations. In addition, high levels of worry are linked to increased use of the third-person perspective (Finnbogadóttir and Berntsen, 2014). In line with the ostensible avoidance function of perspective bias in depression, these findings might suggest that in generalized anxiety disorder (which is characterized primarily by worry), the detached third person perspective complements the tendency toward unproductive, repetitive verbal thought as a means of avoiding affect. However, to date, the majority of relevant studies have focused on *memory* rather than future episodic thinking which is more relevant to generalized and other anxiety disorders.

A number of studies suggested that the effect of instructions to recall emotional events from the third-person perspective on emotion depended on an additional cognitive step. Specifically, the intensity of emotion was lower when an event was recalled from a third-person perspective, and the focus was on *why* the event happened, rather than *what* happened, particularly in studies on anger (Kross et al., 2005; Ayduk and Kross, 2008; Mischkowski et al., 2012; Katzir and Eyal, 2013, Study 1). The “why” manipulations resulted in greater semantic change, which could reflect the greater contextual information offered by a more distanced perspective. These findings may offer a key insight into the difference between the adoption of the third-person perspective as an avoidance strategy compared to one which promotes effective emotion-regulation. When used to promote *avoidance* of negative affect, an often counter-productive emotion-regulation strategy (Hayes et al., 2011), the third-person perspective may not be accompanied by the additional cognitive operations (explicitly simulated by asking “why” questions in the studies by Kross and colleagues) which can introduce new, contextual information. Rather, use of the third-person perspective as an avoidance strategy may reflect a static, inflexible cognitive style, which precludes semantic change.

The pattern of lower levels of affect with a third-person perspective was also seen in most of the studies relating to positive affect associated with autobiographical memories and imagined scenarios (Holmes et al., 2008; Gruber et al., 2009; Vella and Moulds, 2014). An absence of this pattern (Nelis et al., 2012) may have been due to not giving consideration to the role of depressive symptoms in *initial* recall perspective of positive memories (Nelis et al., 2013).

The discussion so far has focused on basic, non-*self* -evaluative emotions. When studies addressing self-conscious emotions are considered, instructions to adopt the third-person perspective was not consistently associated with reduced negative affect, with two studies showing no decrease (Katzir and Eyal, 2013) and another, an increase (Hung and Mukhopadhyay, 2012). In the case of emotions that involve self-evaluation, self-esteem may be an important factor in whether the third-person perspective increased or decreased shame (Libby et al., 2011). Libby and colleagues link this to the influence of “self-defeating interpretive frameworks” (p. 1171); this implies that the context within which the self is evaluated interacts with perspective and this may explain the discrepancies observed in the studies on self-conscious emotions.

The original (spontaneously adopted) perspective of a memory may have a role in determining whether affect intensity changes, as shown by those studies in which the perspective assigned is a shift from the original perspective. All such studies reviewed here found a reduction in intensity of affect when shifting from an original first-person perspective to the third-person, but no difference with the converse shift. Yet, few studies identified in this review established the original perspective associated with the memories, prior to instructing the recall perspective. If the above pattern is generally true, it may be the case that when participants are instructed to take a third-person perspective regardless of spontaneous perspective, changes in affect tend to be due to changes from the more prevalent first-person memories (Nigro and Neisser, 1983). Participants who spontaneously adopted a third-person perspective at recall, regardless of subsequent perspective manipulation, would therefore not be expected to show a reduction in affect.

Further, if the biased adoption of the third-person represents an avoidance strategy, it raises a clinically relevant question as to whether emotional processing could be facilitated through a shift in perspective from third- to first-person.

Future research in this area would benefit from greater methodological clarity and detail. Firstly, in studies examining the effects of perspective on affect, the nature of participants' baseline (i.e., “preferential” or spontaneous) perspective should be routinely assessed. Secondly, assessment of affective states should be performed using validated instruments (e.g., the iPANAS for general affect or suitably brief emotion-specific measures) rather than unvalidated single-item measures. Further, given the intriguing finding that semantic change is a mediator of affective change it is of interest to determine whether “why analysis” manipulations during perspective shifts promote semantic change across emotion categories (particularly anxiety and sadness) and participant groups (non-clinical and clinical). Since “why?” questions can promote an abstract processing style characterized by unproductive repetitive thinking (rumination) in depression (Watkins and Teasdale, 2001) it would be particularly interesting to determine the effects of structured and time-limited “why?” analyses (as used in studies by Kross and colleagues) in the context of perspective shifting in clinical depression for example.

Given the apparent contrast between third-person perspective as an effective emotion-regulation strategy and as characteristic of those with mood or anxiety disorders, future studies should investigate the effect of perspective change within clinical populations; although some work has already started in this area (Kross et al., 2012), it remains unclear as to the effect in anxious populations for example. A more comprehensive understanding of the extent and role of spontaneous and strategic third person perspective deployment across disorders seems appropriate. To further clarify the nature of the relationship between perspective change and avoidance as a cognitive style, measures of avoidance should be employed, for both autobiographical memories (e.g., Horowitz et al., 1979) and future episodic thinking (e.g., Deeprose and Holmes, 2010).

Nonetheless, clinicians should still carefully examine the perspective adopted in imagery to consider its impact on a client's experience of affect (Hales et al., 2014). This review suggests that, for autobiographical imagery at least, the perspective adopted in the image could have important emotional consequences, and if the same pattern is present in the type of intrusive imagery experienced in clinical disorders (Pearson et al., 2015), this could represent a target for intervention.

Finally, it would be helpful if future studies could clarify how strategic change in perspective compares against other emotion regulation strategies. For example, given the link between imagery and affect (Holmes and Mathews, 2005, 2010), it would be of interest to determine the relative efficacy of visuospatial (perspective change) and verbal reappraisal as emotion regulation strategies.

We acknowledge this review has some limitations in terms of methodology and scope. For example, only one author identified articles, and this may have resulted in inadvertent exclusions. Further, the scope of the review was limited to first- and third-person perspectives. However, we acknowledge that other types of spatiotemporal perspective manipulation can impact affect, for example, imagined increasing spatial distance between a recalled scene and the observer (perspective). When negative scenes are imagined as moving away, this is linked to lower negative affect (Davis et al., 2011). In addition to this, *temporal* distance has been shown to interact with vantage perspective (D'Argembeau and Van der Linden, 2004, 2012). For example, memories and imagined future events which are further away temporally are more likely to be experienced from a third-person perspective, compared to those which are nearer temporally being experienced from a first-person perspective D'Argembeau and Van der Linden (2004). Finally, our search terms may have failed to identify some studies on prospection given that we did not use terms relating to “episodic foresight.”

To summarize, the perspective adopted by individuals, whether that is a detached third-person perspective, or a first-person perspective can have important implications for the experience and management of affect in non-clinical populations, and potentially clinical populations too. It may be the case that in some cases, a shift to the third-person perspective aids emotional-regulation in the short term, but for longer term adaptive processing, new information also needs to be incorporated.

## Author contributions

SWH designed and conducted the systematic review; SWH and SK wrote the article for publication.

## Funding

This research was conducted at and funded entirely by University College London.

### Conflict of interest statement

The authors declare that the research was conducted in the absence of any commercial or financial relationships that could be construed as a potential conflict of interest.

## Footnotes

^1^It should be noted that studies in this area use a multiplicity of terms to describe the same phenomena. Studies may refer to, for example, first vs. third-person, field vs. participant/observer, or distanced vs. immersed perspectives. Here, the terms first- and third-person perspective will be adopted throughout for consistency.

## Appendix 1: literature review search terms

### Web of science search terms

“Vantage Perspective” OR “Vantage point” OR “Field perspective” OR “Observer perspective” OR “Observer's perspective” OR “Field-observer perspective” OR “Observer perspective-taking” OR “First person perspective” OR “1st person perspective” OR “Third person perspective” OR “3rd person perspective” OR “Perspective taking” OR “Psychological distanc∗” OR “Visual perspective” OR “Self distanc∗” OR “Self immer∗.”

#### AND

Emotion∗ OR “psychological well-being” OR “psychological wellbeing” OR Stress∗ OR Trauma∗ OR Anxiety OR anxious OR Depress∗ OR Dysphori∗ OR Anger OR angry OR phobi∗ OR fear OR disgust OR worry.

#### AND

“Self-imag∗” OR “Mental Image∗” OR Image∗ OR Imagin∗ OR Memor∗ OR “Verbal process∗” OR “Self-talk∗” OR “Self-evaluat∗” OR Analy∗ OR Ruminat∗ OR avoid∗ OR evaluat∗ OR repetitive OR intru∗ OR future OR prospect∗.

### PsychInfo search terms

1. (Vantage adj Perspective)
2. (Vantage adj point)
3. (Field adj perspective)
4. (Observer adj perspective)
5. (“Observer's” adj perspective)
6. (Field-observer adj perspective)
7. (Observer adj perspective-taking)
8. (First adj (person adj perspective))
9. (1st adj (person adj perspective))
10. (Third adj (person adj perspective))
11. (3rd adj (person adj perspective))
12. (Perspective adj taking)
13. (Psychological adj distanc∗)
14. (Visual adj perspective)
15. Self-distanc∗
16. Self-immer∗
17. 1 or 2 or 3 or 4 or 5 or 6 or 7 or 8 or 9 or 10 or 11 or 12 or 13 or 14 or 15 or 16
18. emotion∗
19. ((Psychological adj well-being) or (Psychological adj wellbeing))
20. Stress∗
21. Trauma∗
22. (Anxiety or anxious)
23. Depress∗
24. Dysphori∗
25. (Anger or angry)
26. Fear
27. Phobi∗
28. Disgust∗
29. Worry
30. exp Emotional Regulation/
31. exp Emotions/
32. exp Emotional Disturbances/
33. exp mental disorders/
34. 18 or 19 or 20 or 21 or 22 or 23 or 24 or 25 or 26 or 27 or 28 or 29 or 30 or 31 or 32 or 33
35. (self adj image∗)
36. (mental adj image∗)
37. image∗
38. imagin∗
39. memor∗
40. exp Memory/
41. exp Imagination/
42. exp Self Concept/
43. (verbal adj process∗)
44. self-talk∗
45. self-evaluat∗
46. Analy∗
47. ruminat∗
48. avoid∗
49. evaluat∗
50. evaluat∗
51. repetitive
52. intrus∗
53. future
54. prospect∗
55. exp Cognitive Processes/
56. exp Self Talk/
57. exp Self Evaluation/
58. 35 or 36 or 37 or 38 or 39 or 40 or 41 or 42 or 43 or 44 or 45 or 46 or 47 or 48 or 49 or 50 or 51 or 52 or 53 or 54 or 55 or 56 or 57
59. 17 and 34 and 58
60. limit 59 to (human and english language and journal article and yr = “1980 –Current”)

### Medline search terms

1. (Vantage adj Perspective)
2. (Vantage adj point)
3. (Field adj perspective)
4. (Observer adj perspective)
5. (“Observer's” adj perspective)
6. (Field-observer adj perspective)
7. (Observer adj perspective-taking)
8. (First adj (person adj perspective))
9. (1st adj (person adj perspective))
10. (Third adj (person adj perspective))
11. (3rd adj (person adj perspective))
12. (Perspective adj taking)
13. (psychological adj distance)
14. (Visual adj perspective)
15. “Self-distanc$”
16. (Self adj immer$)
17. 1 or 2 or 3 or 4 or 5 or 6 or 7 or 8 or 9 or 10 or 11 or 12 or 13 or 14 or 15 or 16
18. Emotion$
19. psychological well-being
20. psychological well-being
21. stress$
22. Trauma$
23. (anxiety or anxious)
24. Depress$
25. Dysphori$
26. (Anger or angry)
27. phobi$
28. fear
29. disgust$
30. worry
31. exp Emotions/
32. exp Mental Disorders/
33. exp Depression/
34. exp Stress, Psychological/
35. exp Affective symptoms/or exp aggression/
36. exp Mental Health/
37. 18 or 19 or 20 or 21 or 22 or 23 or 24 or 25 or 26 or 27 or 28 or 29 or 30 or 31 or 32 or 33 or 34 or 35 or 36
38. Self-imag$
39. (Mental adj Image$)
40. Image$
41. Imagin$
42. memor$
43. exp Memory/
44. exp Imagination/
45. exp “Imagery (Psychotherapy)”/
46. (Verbal adj process$)
47. Self-talk$
48. Self-evaluat$
49. Analy#$
50. Ruminat$
51. avoid$
52. evaluat$
53. repetitive
54. intru$
55. future
56. prospect$
57. exp Self Concept/
58. exp Cognition/
59. exp Communication/
60. 38 or 39 or 40 or 41 or 42 or 43 or 44 or 45 or 46 or 47 or 48 or 49 or 50 or 51 or 52 or 53 or 54 or 55 or 56 or 57 or 58 or 59
61. 17 and 37 and 60
62. limit 61 to (english language and humans and yr = “1980 –Current” and journal article)
63. (psychological adj distanc$)
64. 17 or 63
65. 37 and 60 and 64
66. limit 65 to (english language and humans and yr = “1980 –Current” and journal article)

### EMBASE search terms

1. (Vantage adj Perspective)
2. (Vantage adj point)
3. (Field adj perspective)
4. (Observer adj perspective)
5. (Field-observer adj perspective)
6. (Observer adj perspective-taking)
7. (First adj (person adj perspective))
8. (1st adj (person adj perspective))
9. (Third adj (person adj perspective))
10. (3rd adj (person adj perspective))
11. (perspective adj taking)
12. (Psychological adj distance)
13. (visual adj perspective)
14. Self-distanc$
15. (Self adj immer$)
16. 1 or 2 or 3 or 4 or 5 or 6 or 7 or 8 or 9 or 10 or 11 or 12 or 13 or 14 or 15
17. Emotion$
18. ((Psychological adj well-being) or (Psychological adj wellbeing))
19. stress$
20. Trauma$
21. (anxiety or anxious)
22. Depress$
23. Dysphori$
24. (Anger or angry)
25. phobi$
26. fear
27. disgust$
28. worry
29. exp emotion/
30. exp mental disease/
31. exp emotionality/
32. exp mental health/
33. exp stress/
34. exp social stress/
35. (Psychological adj distanc$)
36. 16 or 35
37. 17 or 18 or 19 or 20 or 21 or 22 or 23 or 24 or 25 or 26 or 27 or 28 or 29 or 30 or 31 or 32 or 33 or 34
38. Self-imag$
39. (Mental adj Image$)
40. Image$
41. Imagin$
42. memor$
43. exp thinking/
44. exp cognition/
45. (Verbal adj process$)
46. Self-talk$
47. self-evaluat$
48. Analy#$
49. Ruminat$
50. avoid$
51. evaluat$
52. repetitive
53. intrus$
54. future
55. prospect$
56. exp verbal behavior/
57. exp self concept/
58. 38 or 39 or 40 or 41 or 42 or 43 or 44 or 45 or 46 or 47 or 48 or 49 or 50 or 51 or 52 or 53 or 54 or 55 or 56 or 57
59. 36 and 37 and 58
60. limit 59 to (human and english language and yr = “1980-Current” and journal)
