# Supplementary material for: The Impact of Perspective Change As a Cognitive Reappraisal Strategy on Affect: A Systematic Review
Source: Front Psychol. 2016 Nov 4;7:1715. doi: 10.3389/fpsyg.2016.01715 (PMC5095639; doi:10.3389/fpsyg.2016.01715)
Supplement: Supplementary file 1 [file Table1.doc]

Table 1.

*Characteristics of the 40 included studies*

| **Study** | **Participants** | **Primary manipulation** | **Conditions** | **Outcome measures** | **Main findings** |
| --- | --- | --- | --- | --- | --- |
| **Dysphoria/ sadness, *n* = 6** | | | | | |
| Grisham et al | Non-clinical student sample | Autobiographical memory: sad experience | 1. First-person (“rumination”) perspective (*n* = 40) 2. Third-person (“reappraisal”) perspective (*n* = 41) | Negative emotion (composite of six emotion words, 0-4 scale)  Positive emotion (composite of five emotion words, 0-4 scale) | Third- compared to first-person perspective  Negative emotion at recall ↓  Positive emotion at recall *↑* |
| Kross & Ayduk , Study 1 | Non-clinical student sample | Autobiographical memory: depressive experience | 1. First-person (“immersed”) reflective analysis of experience (*n =* 48) 2. Third-person (“distanced”) reflective analysis of experience (*n* = 48) 3. Distraction, thinking about affectively neutral information *(n* = 45) | Negative affect index calculated from the valence subscale of the SAM and ratings of “sadness” and “depression” (1-5 scale). | Third- compared to first-person perspective  Negative affect ↓  Third-person compared to distraction  Negative affect  *↔*  First-person compared to distraction  Negative affect  *↑* |
| Kross and Ayduk , Study 2 | Non-clinical student sample | Autobiographical memory: depressive experience | 1. First-person (“immersed”) reflective analysis of depressive experience, with 1 day follow-up (*n* = 56) and 7 day follow up (*n* = 40) 2. Third-person (“distanced”) reflective analysis of depressive experience with 1 day follow-up (*n* = 57) and 7 day follow up (*n*= 38) 3. Distraction, thinking about affectively neutral information with 1 day follow-up (*n* = 58) and 7 day follow up (*n =* 37) | Negative affect index calculated from the valence subscale of the SAM and ratings of “sadness” and “depression” (1-5 scale). | Third- compared to first-person perspective  Negative affect ↓  Third-person compared to distraction  Negative affect  *↔*  First-person compared to distraction  Negative affect  *↑*  *At follow-up (1/7 day)*  Third- compared to first-person perspective/distraction  Negative affect ↓  First-person compared to distraction  Negative affect  *↔* |
| Kross et al | Clinical sample (Adults with major depressive disorder; MDD) and healthy control group | Autobiographical memory: sad memory | 1. First-person (“immersed”) reflective analysis of sadness experience, (*n* = 25 MDD, *n* = 21 control) 2. Third-person (“distanced”) reflective analysis of sadness experience (*n* = 26 MDD, *n* = 24 control) | Negative affect: SAM  Negative affect index calculated from the SAM and the Negative scale of the PANAS. | Third-person compared to first-person perspective: MDD  Negative affect index ↓  Third-person perspective: MDD  Baseline-post negative affect ↓ (trend)  First-person perspective: MDD  Baseline-post negative affect *↑*  First- and third-person perspective: Control  Negative affect *↔* |
| Williams and Moulds | Dysphoric subclinical sample | Autobiographical memory: intrusive negative memory | 1. Spontaneously adopted (at recall) first-person perspective “negative intrusive memories” recalled from the third-person perspective (*n =* 78) 2. Spontaneously adopted (at recall) third-person perspective “negative intrusive memories” recalled from the first-person perspective (*n =* 55) | Distress (0-100 scale)  Richness of emotion recollection (1-7 scale)  Anxiety at recollection (1-7 scale) | Changing from a first- to a third-person perspective  Richness of emotion *↔*  Distress ↓  Anxiety compared to converse shift ↓  Changing from a third- to a first-person perspective  Richness of emotion *↔*  Levels of distress *↔*  Anxiety compared to converse shift *↑* |
| Wisco & Nolen-Hoeksema | Subclinical and control groups (High (≥ 16 BDI-II) or low (≤ 9 BDI-II) dysphoria) | Autobiographical memory: depression experience  Imagined scenarios: ambiguous scenario testing imagery interpretation bias | 1. First-person (“immersed”) reflective analysis of depression experience, and of guided imagery of ambiguous situations   (*n* = 28 non-dysphoric, *n* = 25 dysphoric)   1. Third-person (“distanced”) reflective analysis of depression experience, and of guided imagery of ambiguous situations   (*n* = 30 non-dysphoric, *n* = 27 dysphoric) | State negative emotion calculated from ratings of sadness and depression (1-9 scale)  Emotion re-experiencing extent  Emotion re-experiencing intensity | Third- and first-person perspective: memory (both groups)  State negative emotion pre/post task *↑*  Third- compared to first-person perspective: memory (both groups)  Re-experiencing intensity ↓  Re-experiencing extent *↔*  First- and third-perspective: guided imagery (both groups)  State negative emotion pre/post task *↑*  Valence of interpretation *↔* |
| **Anxiety/threat, *n* = 6** | | | | | |
| Kross et al , Study 2 | Non-clinical female student sample | Mood induction: verbal “self-talk” in a social evaluation task. | 1. First-person pronoun reflective analysis of feelings (*n* = 44) 2. Third-person (“non-first”) pronoun analysis of feelings (*n* = 45) | Anxiety (1-5 scale) | Third- compared to first-person perspective  Pre-task anxiety *↔*  Post-task anxiety faster ↓ |
| Kross et al , Study 3 | Non-clinical student sample | Mood induction: verbal “self-talk” in a social stress induction task | 1. First-person pronoun reflective analysis of feelings (*n* = 45) 2. Third-person (“non-first”) pronoun reflective analysis of feelings (*n* = 44) | Negative affect (1-7 scale)  Post-task shame  Shame and Pride subscale of State Shame and Guilt Scale. | Third-person perspective  Negative affect pre-post *↔*  First-person perspective  Negative affect pre-post task *↑*  Third- compared to first-person perspective  Post-task shame ↓ |
| Kross et al , Study 4 | Non-clinical student sample | Mood induction: verbal “self-talk” in a social stress induction task | 1. First-person pronoun reflective analysis of feelings (*n* = 37) 2. Third-person (“non-first”) pronoun reflective analysis of feelings (*n* = 38) | Anticipatory anxiety (1-7 scale)  Task threat (1-7 scale)  Task challenge (1-7 scale) | Third- compared to first-person perspective  Anticipatory anxiety ↓  Task threat ↓  Task challenge *↑* |
| Lau et al | Non-clinical student sample | Mood induction: recall of inclusion or ostracism experience | 1. First-person perspective recall of either inclusion or ostracism experience 2. Third-person perspective recall of either inclusion or ostracism experience   *N* per condition not reported. Sample *n =* 56, approx. *n* = 14 per group. | Threatened needs  PNQ including self-esteem, belonging and meaningful existence. | Third- compared to first-person perspective (ostracism group)  Threatened needs at time 1 *↔*  Threatened needs at time 2 *↑*  First-/third-person perspective (inclusion group)  Threatened needs at time 1 and 2 *↔* |
| Spurr & Stopa | Subclinical and control groups (high and low social anxiety) | Mood induction: speech task | Within-subjects design   1. First-person perspective adopted while giving filmed presentation 2. Third-person perspective adopted while giving filmed presentation   Order counterbalanced (*n* = 22 high anxiety, *n* = 22 low anxiety). | Anxiety measured pre- and post each presentation (0-100 scale). | Third- compared to first-person perspective (both groups)  Anxiety *↑* (trend) |
| Wang et al | Internet-based sample of Taiwan residents, high and low anxious or avoidant attachment | Mood induction: writing about a conflict scenario to induce threat | 1. Writing from first-person perspective (*n* = 40) 2. Writing from third-person perspective (*n* = 43) | Negative emotion calculated from eight aggregated negative emotion scores, e.g. fear and hostility (1-7 scale).  Positive emotion calculated from six aggregated positive emotion scores, e.g. peace and happiness (1-7 scale). | Third- compared to first-person perspective  Negative emotion ↓  Positive emotion *↔*  Third-person perspective in low compared to high avoidant attachment  Negative emotion ↓  Positive emotion *↑*  First-person perspective in low compared to high avoidant attachment  Negative emotion *↔*  Positive emotion *↔*  Third- compared to first-person perspective in low and high anxious attachment  Negative emotion ↓  Positive emotion *↑* (low anxious attachment only) |
| **Anger, *n* = 6** | | | | | |
| Ayduk & Kross | Non-clinical student sample | Autobiographical memory: feeling anger | 1. First-person (“self-immersed”) “why” reflective analysis 2. Third-person (“self-distanced”) “why” reflective analysis   *N* per condition not reported. Sample *n =* 84 after exclusions, approx. *n* = 42 per group. | Emotional reactivity index computed from extent to which and intensity with which original emotions were experienced (1-7 scale). | Third- compared to first-person perspective  Emotional reactivity ↓ |
| Kross et al , Study 1 | Non-clinical student sample | Autobiographical memory: feeling anger/hostility | 1. First-person perspective “what” focus on memory (*n* = 38) 2. First-person perspective “why” focus (*n* = 39) 3. Third-person perspective “what” focus on memory (*n* = 39) 4. Third-person perspective “why” focus on memory (*n* = 39) | Implicit anger measured through a word completion task.  Negative affect measured by the Negative Affect subscale of the PANAS.  Explicit anger index computed from responses to ‘hostile’ and ‘irritable’ in PANAS. | Third-person “why” perspective compared to first-person “why” and first/third-person “what” perspectives  Implicit anger ↓  Negative affect ↓  Explicit anger ↓ |
| Kross et al , Study 2 | Non-clinical student sample | Autobiographical memory: feeling anger/hostility | 1. First-person perspective “why” focus on memory 2. Third-person perspective “why” focus on memory   *N* per condition not reported. Sample *n =* 133, approx *n* = 66 per group. | Emotional reactivity index computed from extent to which and intensity with which original emotions were experienced (1-7 scale). | Third- compared to first-person perspective  Emotional reactivity ↓ |
| Mischkowski et al , Experiment 1 | Non-clinical student sample | Mood induction: anger | 1. First-person perspective (*n* = 28) 2. Third-person perspective (*n* = 30) 3. No perspective manipulation control (*n* = 36) | Implicit aggression measured through a word completion task.  Anger index computed from the valence subscale of the SAM and four emotion word ratings (e.g. angry/hostile; 1-5 scale). | Third- compared to first-person perspective and control  Implicit aggression ↓  Anger ↓ |
| Ray et al | Non-clinical student sample, all female | Autobiographical memory: anger | 1. First-person (“rumination”) perspective (*n* = 34) 2. Third-person (“reappraisal”) perspective (*n* = 43) | Anger (0-4 scale)  Negative emotion computed from composite of six emotion words (0-4 scale).  Positive emotion computed from composite of five emotion words (0-4 scale). | Third- compared to first-person perspective  Anger at recall ↓  Anger after rest *↔*  Negative emotion at recall and after rest ↓  Positive emotion at recall and after rest *↔* |
| Wimalaweera & Moulds | Non-clinical student sample | Autobiographical memory: anger in past 12 months | 1. First-person perspective “what” focus on memory 2. First-person perspective “why” focus on memory 3. Third-person perspective “what” focus on memory 4. Third-person perspective “why” focus on memory   *N* per condition not reported. Sample *n =* 60 after exclusions, approx. *n* = 15 per group. | Implicit anger measured through a word completion task.  Explicit anger computed from items (e.g. “anger/hostile/irritable”) from the PANAS (expanded form).  Negative affect subscale of the PANAS (expanded form). | Third-person “why” perspective compared to first-person “why” and first/third-person “what” perspectives  Implicit anger *↑*  First-person “what” and “why, and third-person “why” perspectives  Explicit anger pre/post task *↑*  Third-person “what” perspective  Explicit anger pre/post task *↔*  First- and third-person “why” perspectives  Negative affect pre/post task *↑*  First- and third-person “what” perspectives  Negative affect pre/post task *↔* |
| **Self-conscious emotions, *n* = 7** | | | | | |
| Hung & Mukhopadhyay , Study 1 | Non-clinical student sample | Imagining scenarios: “self-control” scenario of either accepting or declining a party invitation before an exam | 1. First-person perspective of self-control 2. Third-person perspective of self-control 3. First-person perspective of non self-control 4. Third-person perspective of non self-control   *N* per condition not reported. Sample *n =* 288, approx. *n* = 72 per group. | Negative ‘basic’ emotion computed from average of ratings of “sad”, “distressed” and “sorrowful” (1-4 scale).  Positive ‘basic’ emotion computed from average of ratings of “excited” and “joyful” (1-4 scale).  Negative self-conscious emotion computed from rating of “guilty” (1-4 scale).  Positive self-conscious emotion computed from average of ratings of “proud” and “relieved” (1-4 scale). | Third- compared to first-person perspective (self-control)  Negative basic emotion ↓  Positive basic emotion *↔*  Negative self-conscious emotion *↔*  Positive self-conscious emotion *↑*  Third- compared to first-person perspective (non self-control)  Positive basic emotion ↓  Negative basic emotion *↔*  Negative self-conscious emotion *↑*  Positive self-conscious emotion *↔* |
| Hung & Mukhopadhyay , Study 2 | Non-clinical student sample | Autobiographical memory: resisting or succumbing to temptation | 1. First-person perspective in recall of resisting temptation 2. Third-person perspective in recall of resisting temptation 3. First-person perspective in recall of succumbing to temptation 4. Third-person perspective in recall of succumbing to temptation   *N* per condition not reported. Sample *n =* 75, approx. *n* = 18 per group. | Sad (1-7 scale)  Guilt (1-7 scale)  Pride (1-7 scale)  Excitement (1-7 scale)  Embarrassment (1-7 scale) | Third- compared to first-person perspective (resist)  Sad ↓  Guilt *↔*  Pride *↑*  Excitement *↔*  Embarrasment *↔*  Third- compared to first-person perspective (succumb)  Guilt *↑*  Sad *↔*  Pride *↔*  Excitement ↓  Embarrasment *↔* |
| Hung & Mukhopadhyay , Study 3 | Non-clinical student sample | Imagining scenarios; embarrassing but exciting scenario | 1. First-person perspective of imagined exciting/embarrasing experience 2. Third-person perspective of imagined exciting/embarrasing experience   *N* per condition not reported. Sample *n =* 26, approx. *n* = 13 per group. | Excitement (1-7 scale)  Embarrassment (1-7 scale)  Sad (1-7 scale)  Guilt (1-7 scale)  Pride (1-7 scale) | Third- compared to first-person perspective  Excitement ↓  Embarrasment *↑*  Sad *↔*  Guilt *↔*  Pride *↔* |
| Katzir & Eyal , Study 1 | Non-clinical student sample | Autobiographical memory: anger or guilt | 1. First-person perspective “why” focus on anger 2. First-person perspective “why” focus on guilt 3. Third-person perspective “why” focus on anger 4. Third-person perspective “why” focus on guilt   *N* per condition not reported. Sample *n =* 241 after exclusions, approx. *n* = 60 per group. | Emotional reactivity index computed from extent to which, and intensity with which original emotions were experienced (1-9 scale). | Third- compared to first-person perspective  Anger ↓  Guilt *↔* |
| Katzir & Eyal , Study 2 | Non-clinical student sample | Imagining scenarios: possible future failure scenarios | 1. First-person perspective “why” focus on sadness 2. First-person perspective “why” focus on shame 3. Third-person perspective “why” focus on sadness 4. Third-person perspective “why” focus on shame   *N* per condition not reported. Sample *n =* 254 after exclusions, approx. *n* = 63 per group. | Emotional reactivity index computed from extent to which and intensity with which original emotions were experienced (1-9 scale). | Third- compared to first-person perspective  Sadness ↓  Shame *↔* |
| Libby et al , Study 4 | Subclinical student sample (high to low self-esteem) | Autobiographical memory: failure, success and neutral experiences | 1. Imagining scenarios from a first-person perspective (*n* = 33) 2. Imagining scenarios from a first-person perspective (*n* = 33) | Shame (guilt-free) calculated from the TOSCA | Third-person perspective  Self-esteem negatively correlated with shame  First-person perspective  No relationship between self-esteem and shame |
| Libby et al , Study 5 | Subclinical student sample (high to low self-esteem, LSE) | Imagining scenarios: possible future failure scenarios | 1. First-person perspective recall of failure experience 2. Third-person perspective recall of failure experience 3. First-person perspective recall of success experience 4. Third-person perspective recall of success experience 5. First-person perspective recall of neutral experience 6. Third-person perspective recall of neutral experience   *N* per condition not reported. Sample *n =* 128 after exclusions, approx. *n* = 21 per group. | Shame (guilt-free) calculated from “ashamed” and “guilty” items of the PANAS. | Third- compared to first-person perspective (failure memory)  Self-esteem negatively correlated with shame  LSE, Shame *↑*  HSE, Shame ↓  Third- compared to first-person perspective (success and neutral memories)  No relationship between self-esteem and shame |
| **Mixed and neutral affect autobiographical and episodic memories, n = 9** | | | | | |
| Bagri and Jones , Experiment 1 | Non clinical student sample | Autobiographical memory: Recall task of novel scenario descriptions | 1. Recall from a first-person perspective (*n* = 14) 2. Recall from a third-person perspective (*n* = 14) | Emotional richness of memory recall  Recall of affective detail | Third- compared to first-person perspective  Emotional richness *↔*  Recall for affective detail ↓ |
| Bagri and Jones , Experiment 2 | Non clinical student sample | Autobiographical memory: Recall task of novel scenario descriptions | Within-subjects design   1. Recall from first- and then third-person perspective or vice versa (*n =* 39)   Order counterbalanced | Emotional richness of memory recall  Recall of affective detail | Third- compared to first-person perspective  Emotional richness ↓  Recall for affective detail ↓ |
| Berntsen & Rubin | Non-clinical  student sample | Autobiographical memory: positive and negative | 1. Recall of memories from an instructed first-person perspective, followed by a switch to third-person perspective for two memories (*n* = 40) 2. Recall of memories from an instructed third-person perspective, followed by a switch to first-person perspective for two memories (*n* = 42) 3. Recall of memories from spontaneously adopted perspective, followed by a switch to opposite perspective (first and third) for two memories (*n* = 40) | Emotional intensity (1-7 scale) | Third- compared to first-person recall (instructed and spontanously adopted)  Emotional intensity *↔*  Third- to first-person perspective shift  Emotional intensity *↔*  First- to third-person perspective shift  Emotional intensity ↓ |
| Crawley | Non-clinical student/  volunteer sample | Autobiographical memory: an unpleasant event recalled from a spontaneously adopted first-person perspective | 1. First-person perspective, writing once about the memory from same perspective. 2. First-person perspective, writing three times about the memory from same perspective. 3. First-person perspective, writing once about the memory from same perspective, and then twice from a third-person perspective.   *N* per condition not reported. Sample *n =* 90, approx. *n* = 30 per group. | Emotion at time of event (1-7 scale):  Negative  Intensity  Physical/bodily  Positive  Emotion at recall (1-7 scale):  Negative  Intensity  Physical/bodily  Positive | *At time of event:*  Repeated third-person perspective  Negative ↓  Intensity ↓  Physical/bodily ↓  Positive *↔*  Repeated first-person perspective  Negative *↑*  Intensity *↔*  Physical/bodily ↓  Positive *↔*  *At recall:*  Repeated third-person perspective  Negative ↓  Intensity ↓  Physical/bodily ↓  Positive *↑*  Repeated first-person perspective  Negative ↓  Intensity ↓  Physical/bodily ↓  Positive *↔* |
| Robinson & Swanson , Experiment 2 | Non-clinical student sample | Autobiographical memories | 1. Twelve autobiographical memories (both perspectives) recalled from a first-person perspective 2. Twelve autobiographical memories (both perspectives) recalled from a third-person perspective   *N* per condition not reported. Sample *n =* 56, approx. *n* = 28 per group. | Original intensity (1-7 scale)  Current intensity (1-7 scale)  Current affect awareness (remembering feelings at time; 1-7 scale)  Original pleasantness (1-7 scale)  Current pleasantness (1-7 scale) | Third- to first-person perspective shift  Original and current intensity *↔*  Current affect awareness *↔*  Original pleasantness *↔*  Current pleasantness *↔*  First- to third-person perspective shift  Original and current intensity ↓  Current affect awareness ↓  Original pleasantness *↔*  Current pleasantness *↔* |
| Seih et al , Study 1 | Non-clinical student sample | Autobiographical memory: an upsetting memory | 1. First-person perspective (“I”) 2. Second-person perspective (“you”) 3. Third-person perspective (“he/she”)   *N* per condition not reported. Sample *n =* 55, approx. *n* =18 per group. | Emotional involvement overall (1-7 scale)  Emotional involvement post-writing (1-7 scale)  Positive affect calculated from the mean of 5 positive items (1-5 scale).  Negative affect calculated from the mean of 6 negative items (1-5 scale). | Third- compared to first- and second-person perspectives  Emotional involvement overall ↓  Emotional involvement post-writing ↓ (trend)  Positive affect *↔*  Negative affect *↔* |
| Sekiguchi & Nonaka | Non-clinical student sample | Autobiographical memory: positive and negative | 1. Recall of memories from spontaneously adopted perspective (first/third) at time 1 (T1), time 2 (T2) and with no perspective instructions at time 3 (T3). 2. Recall of memories from spontaneously adopted perspective (first/third) at T1, the opposite perspective at T2, and with no perspective instructions at T3.   *N* per condition not reported. Sample *n =* 48, approx. *n* = 24 per group. | Emotional intensity (1-5 scale) | Third- to first-person perspective, at T2 and T3, compared to T1  Emotional intensity *↔*  First- to third-person perspective, at T2 and T3, compared to T1  Emotional intensity ↓ |
| Sutin and Robins , Study 2 | Non-clinical student sample | Autobiographical memory: self-defining memories | 1. Recall from spontaneously adopted perspective (*n* = 450) 2. Recall from instructed first-person perspective (*n* = 128) 3. Recall from instructed third-person perspective (*n* = 128) | Emotion intensity measured on the MEQ. | Third- compared to first-person perspective (spontaneously adopted at initial recall)  Emotion intensity ↓  Third- compared to first-person perspective (instructed)  Emotion intensity *↔* |
| Terry and Horton | Non clinical student sample | Autobiographical memory: unpleasant, emotional and self-conscious memories | Within-subjects design   1. Recall from either first- and then third-person perspective or vice-versa; followed by participant comparison of the two perspectives *(n =* 24)     Order counterbalanced. | Degree of emotion (1-5 scale)  Nervous (1-5 scale)  Self-consciousness (1-5 scale)  Uneasiness (no scale given) | Third- compared to first-person perspective  Degree of emotion ↓  Nervous ↓  Self-consciousness ↓  Participant comparison of third- compared to first-person perspective  Degree of emotion ↓  Nervous ↓  Uneasiness ↓ |
| **Positive affect, *n* = 4** | | | | | |
| Gruber et al | Clinical sample (Adults with bipolar I disorder; BP) and healthy control group | Autobiographical memory: intense happiness | Within-subjects design   1. First-person perspective ‘why’ reflective analysis of memory follow by third-person perspective ‘why’ reflective analysis of memory, or vice versa (counterbalanced)   (*n* = 27 BP, *n* = 27 control) | Positive affect  Positive subscale of the PANAS – Short Form  Number of positive thoughts (1-6 scale) | Third-person compared to first-person perspective (both groups)  Positive affect ↓  Number of positive thoughts ↓ |
| Holmes et al | Non clinical university sample | Imagining scenarios: mental imagery or verbal processing of positive imaginary scenarios | 1. Imagining scenarios from a first-person perspective 2. Imagining scenarios from a third-person perspective 3. Verbal processing of scenarios     *N* per condition not reported. Sample *n =* 78, approx. *n* = 26 per group. | Positive affect  Positive subscale of the PANAS (21 items) | Third-person perspective and verbal condition  Positive affect ↓ *↔*  First-person perspective  Positive affect *↑* |
| Nelis et al | Non-clinical student sample | Imagining scenarios: mental imagery or verbal processing of positive imaginary scenarios | 1. Imagining scenarios from a first-person perspective 2. Imagining scenarios from a third-person perspective 3. Verbal processing of scenarios   *N* per condition not reported. Sample *n =* 78, approx. *n* = 26 per group | Positive affect  PANAS positive subscale (10 items) | Third- and first-person perspective compared to verbal condition  Positive affect *↑*  Third-person compared to first-person perspective  Positive affect *↔* |
| Vella & Moulds | Non-clinical student sample | Autobiographical memory: Positive memory  Imagining scenarios: positive future event | 1. First-person memory recall switching to third-person perspective (*n* = 42) 2. Third-person memory recall switching to first-person perspective (*n* = 32)   And   1. First-person imagined event switching to third-person perspective (*n* = 35) 2. First-person imagined event switching to third-person perspective (*n* = 38) | Happiness (0-100 scale)  Optimism (0-100 scale)  Hopefulness (0-100 scale) | *Memory recall*  Third- to first-person perspective shift  Happiness *↔*  Optimism *↔*  Hopefulness *↔*  First- to third-person perspective shift  Happiness ↓  Optimism ↓  Hopefulness ↓  *Imagined event*  Third- to first-person perspective shift  Happiness *↔*  Optimism *↔*  Hopefulness *↔*  First- to third-person perspective shift  Happiness ↓  Optimism ↓  Hopefulness ↓ (trend) |

*Note*. ↑ = increased levels of affect; ↔ = no significant difference between groups or pre-post; ↓ = decreased levels of affect.

SAM, Self Assessment Mannequin; PANAS, Positive and Negative Affect Schedule; PNQ, Primary Needs Questionnaire; TOSCA, Test of Self-Conscious Affect; MEQ, Memory Experiences Questionnaire
